# Paradoxical changes in mood-related behaviors on continuous social isolation after weaning

**DOI:** 10.1007/s00221-021-06149-x

**Published:** 2021-06-18

**Authors:** Hiyori Matsumoto, Naoto Omata, Yasushi Kiyono, Tomoyuki Mizuno, Kayo Mita, Hirotaka Kosaka

**Affiliations:** 1 Department of Neuropsychiatry, Faculty of Medical Sciences, University of Fukui, 23-3 Matsuokashimoaizuki, Eiheiji-cho, Yoshida-gun, Fukui, 910-1193 Japan; 2 Department of Nursing, Faculty of Health Science, Fukui Health Science University, 55 Egami-cho 13-1, Fukui, 910-3190 Japan; 3 Biomedical Imaging Research Center, University of Fukui, 23-3 Matsuokashimoaizuki, Eiheiji-cho, Yoshida-gun, Fukui, 910-1193 Japan

**Keywords:** Mood, Social isolation, Behavioral change, Puberty, Adulthood, Lithium

## Abstract

Continuous social isolation (SI) from an early developmental stage may have different effects in youth and adulthood. Moreover, SI is reported to impair neuronal plasticity. In this study, we used post-weaning rats to compare the impact of continuous SI on depressive-like, anxiety-related, and fear-related behaviors and neuronal plasticity in puberty and adulthood. Furthermore, we assessed the effect of lithium on behavioral changes and neuronal plasticity. Continuous SI after weaning induced depressive-like behaviors in puberty; however, in adulthood, depressive-like and anxiety-related behaviors did not increase, but—paradoxically—decreased in comparison with the controls. The decreased expression of neuronal plasticity-related proteins in the hippocampus in puberty was more prominent in the prefrontal cortex and hippocampus in adulthood. In contrast, SI after weaning tended to decrease fear-related behaviors in puberty, a decrease which was more prominent in adulthood with increased neuronal plasticity-related protein expression in the amygdala. Lithium administration over the last 14 days of the SI-induced period removed the behavioral and expression changes of neuronal plasticity-related proteins observed in puberty and adulthood. Our findings suggest that the extension of the duration of SI from an early developmental stage does not simply worsen depressive-like behaviors; rather, it induces a behavior linked to neuronal plasticity damage. Lithium may improve behavioral changes in puberty and adulthood by reversing damage to neuronal plasticity. The mechanisms underlying the depressive-like and anxiety-related behaviors may differ from those underlying fear-related behaviors.

## Introduction

Depression is a common mood disorder that raises major public health concerns (Smith 2014) and is influenced by several social problems. Specifically, the relationship between social isolation (SI) and depression has been well investigated. SI has been shown to be associated with depression, even after controlling for age, gender, employment status, and other covariates (Ge et al. 2017). In animal experiments, SI-induced rats have been used as a model of depression. For example, SI-induced rats display prolonged immobility in the forced swim test, indicating an increase in depressive-like behaviors, while also avoiding the open zone in the elevated plus-maze test, indicating an increase in anxiety-related behaviors (Gilles et al. 2017; Skelly et al. 2015).

SI can happen not only in adulthood (after puberty or adolescence) but from an early developmental age as well (after weaning or in early childhood). Recently, depression in youth, i.e., during puberty or adolescence, has attracted attention, and either SI or insufficient attachment in early developmental stages is thought to be related to the onset of depression in puberty or adolescence (Ford and Rechel 2012; Shaw and Dallos 2005). An experiment using animals reported that the limitations of maternal attachment from postnatal day (P) 8 to P12 induced depressive-like behaviors in puberty (Raineki et al 2012). Conversely, it has also been shown that people who had been exposed to long-term SI or neglect from an early developmental age can develop other psychiatric problems, such as violence or aggression (Kalvin and Bierman 2017; Naugton et al 2013). Studies in animals have also demonstrated the induction of hyperactivity or aggressive-like behaviors by extended post-weaning SI (Fabricius et al. 2011; Tulogdi et al. 2014). Therefore, there is a possibility that continuous SI from an early developmental stage has different impacts in youth and adulthood. With continuous SI, depressive symptoms do not simply worsen, but different patterns of emotional changes may occur without the addition of other stresses.

As the main purpose of this study, we evaluated the influence of continuous SI on depressive-like and anxiety-related behaviors in puberty and adulthood in post-weaning rats. Furthermore, fear-related behaviors were also assessed. It is known that SI causes damage to neuronal plasticity (Djordjevic et al. 2012). Therefore, to investigate the clue of the mechanism underlying the behavioral changes that occurred following SI, changes in neuronal plasticity were analyzed by assessing the expression of neuronal plasticity-related proteins. This included brain-derived neurotrophic factor (BDNF), phosphorylated extracellular signal-regulated kinase 1/2 (pERK), phosphorylated cyclic AMP (cAMP) responsive element-binding protein (pCREB), synaptophysin, and post-synaptic density 95 (PSD95). Indeed, BDNF is one of the most representative proteins associated with the regulation of neuronal plasticity, and its transcription is induced by pERK and pCREB (Alonso et al. 2005; Autry and Monteggia 2012; Pruusnild et al. 2011). Pre-synaptic and post-synaptic plasticity and the maturation of synapses are governed by synaptophysin and PSD95, respectively (Fourneau et al. 2018) (Vickers et al. 2006). Both the prefrontal cortex and hippocampus are well known to be associated with the pathophysiology of depression, especially in puberty or adolescence and adulthood (Straub et al. 2019; Wang et al. 2018). Furthermore, the amygdala relates to fear and affective behaviors in an age-dependent manner (Zhang and Rosenkranz 2013). Therefore, the expression of neuronal plasticity-related proteins was evaluated in these brain regions. Lithium has a therapeutic and neurotrophic effect on mood disorders (Won et al. 2017; Kessing et al. 2018) and is also used in animal models of depression (Kin et al. 2019). Therefore, in searching for a way to treat unstable moods associated with continuous SI from an early age, we also investigated the effect of lithium on the behavioral changes and neuronal plasticity.

## Materials and methods

### Animals and experimental protocol

**Fig. 1:**
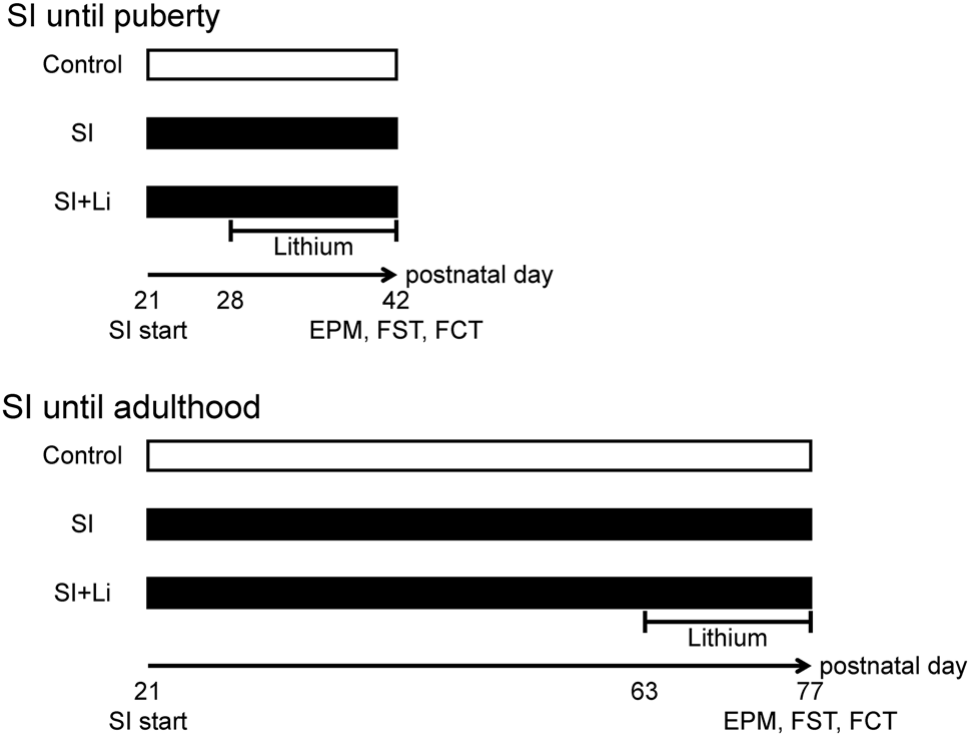
Study protocol. SI: social isolation; Li: lithium; P: postnatal day; EPM: elevated plus-maze test; FST: forced swim test; FCT: fear conditioning test

All experiments were conducted in accordance with the National Institutes of Health’s policy regarding the use of animals in experimental research and were approved by the institutional animal care committee at the University of Fukui. Three-week-old male Wistar rats were purchased from Sankyo Labo Service Corp. (Tokyo, Japan). They were housed under controlled conditions (at a temperature of 24 ± 1 °C) and according to a 12-h light–dark cycle with ad libitum access to food and water. SI was performed by housing each rat individually using an independent rack to prevent any sensory contact with other animals. Figure 1 reveals the protocol of this study. To investigate the effect of continuous SI from weaning in puberty and adulthood, rats were housed for 3 and 8 weeks (P42, early puberty and P77, young adult, respectively) (Ketelslegers et al. 1978; Sengupta 2013), and the rats in the control group were housed in a group (2–3 rats per cage) for the same period as the group of rats in SI. Both the SI until puberty (without lithium) and the control group consisted of 21 animals each, that were used to set up this study's experimental systems. Conversely, the other groups consisted of 9–10 animals for minimizing the sacrificed animal number. To habituate them to human contact, the rats were handled briefly several days per week. After the respective housing time periods, the elevated plus-maze test (EPM) was carried out, after which the forced swim test (FST) was carried out. The fear conditioning test (FCT) was carried out using different rats from those used for the EPM and FST. To assess the effect of lithium on behavioral changes, lithium was administered by changing the drinking water from normal water to lithium-containing water for 14 days before the end of the SI protocol. A lithium solution of 12 mM (for the group of rats in SI until puberty) or 30 mM (for the group of rats in SI until adulthood) was used. Lithium chloride was purchased from Wako Pure Chemical Industries Ltd. (Osaka, Japan). We did nothing to mask the taste of lithium, such as using sucrose because we prevented the inclusion of other factors which may affect the animals’ behaviors. After the behavioral tests, the rats’ body weights were measured (Table 1), and blood samples were collected. These protocols for the administration of lithium resulted in serum concentrations of 0.62 ± 0.28 mEq/l (for the group of rats in SI until puberty) (mean ± standard deviation (SD)) or 0.75 ± 0.20 mEq/l (for the group of rats in SI until adulthood), respectively. These serum concentrations are close to the range that is achieved and maintained during lithium therapy in human subjects (0.4–1.2 mEq/l). It is suggested that the half-life of lithium in rats is shorter than that in humans, but the concentrations of lithium in serum and brain tissue are almost equal in both rat and human (Johnson et al. 1982; Lee et al. 2012; Wraae 1978). Furthermore, lower concentrations of lithium, around 0.7 mEq/L, are often used in young patients (Landersdorfer et al. 2017; Siegel 2014). The expression patterns of neuronal plasticity-related proteins in the brain were evaluated using different rats from those used for the behavioral tests.

**Table 1:** Bodyweight (g) after SI period

	Control	SI	SI + Li
SI until puberty	222.6 ± 13.7	222.0 ± 13.2	176.7 ± 12.7^**, ††^
SI until adulthood	400.0 ± 19.5	412.0 ± 24.4	292.5 ± 19.8^**, ††^

All values are mean ± SD (*n* = 9–21)

*SI* social isolation, *Li* lithium

^**^p < 0.01 compared with control, ^††^*p* < 0.01 compared with SI (social isolation without lithium)

#### EPM

Anxiety-related behaviors were evaluated using the EPM as described previously (Mitsuya et al. 2015; Pellow et al. 1985). Briefly, the plus-shaped apparatus (situated 65 cm above the floor) consisted of two open arms (45 cm × 10 cm) and two closed arms (45 cm × 10 cm, surrounded by 50 cm high non-transparent walls) that extended from a central platform (10 cm × 10 cm). Each individual rat was placed on the central platform facing the closed arms and was allowed to explore the maze freely. The observation lasted 5 min using a video camera set above the EPM apparatus and was analyzed by the ANY-maze video-tracking system (Stoelting Co., Wood Dale, IL, USA). The ratio (%) of the time spent in the open arms to the total time spent in all arms and the total distance travelled were evaluated as an index of anxiety-related behaviors and activity, respectively. In each group, several rats fell off from the open arms and were excluded from the analysis.

#### FST

Depressive-like behaviors were evaluated using the FST as described previously (Mitsuya et al. 2015; Prosolt et al. 1977). Briefly, the day before the test, each rat was placed into a plastic cylinder (height: 80 cm, diameter: 45 cm) filled with water to a 25-cm depth at 25 ± 1 °C for 15 min on the day after the EPM for the habituation session. On the test day, each rat was placed into the cylinder again, and the observation lasted 5 min using a video camera. Immobility time and the total duration of periods of inactivity were independently measured by three people, and the meantime was used and scored as an index of depressive-like behaviors.

#### FCT

Fear conditioning and extinction were evaluated using the FCT as described previously (Zhang and Rosenkranz 2013). Briefly, for conditioning trials, rats were placed in an acrylic chamber (22 × 25 × 48 cm height) with metal grids. The conditioning trials comprised a 120 s habituation followed by five pairings of a neutral tone (10 s, 2900 Hz, 83 dB) with a footshock (1 s, 0.8 mA). Neutral tone co-terminated with the footshock. Conditioning trials were presented at 60 s inter-trial intervals. After 24 h, extinction trials were performed in a visually and tactile different chamber (22 × 25 × 50 cm height) with a planar floor. Extinction trials consisted of 120 s habituation followed by a conditioned tone 15 times without a footshock. Extinction trials were presented at 60 s inter-trial intervals. Behaviors were observed using a video camera set above the FCT apparatus and were analyzed by the ANY-maze video-tracking system. The ratio (%) of the time spent freezing from trial to trial was assessed as an index of fear-related behaviors.

### Western blot analysis

The prefrontal cortex, hippocampi, and amygdala were isolated from rats in each group and homogenized using BioMasher (Nippi Research Institute of Biomatrix, Ibaraki, Japan) with RIPA buffer (10 mM Tris–HCl pH 7.4, 150 mM NaCl, 1 mM EDTA, 1% NP40, 0.1% sodium dodecyl sulfate (SDS), 0.1% Na deoxycholate), including a protease inhibitor cocktail (Nacalai Tesque, Kyoto, Japan) and a phosphatase inhibitor cocktail (Nacalai Tesque, Kyoto, Japan). A mass of 20 µg of the total proteins was separated by SDS–polyacrylamide gel electrophoresis (10–20%) and transferred to polyvinylidene difluoride membranes. After being blocked with EzBlockChemi (ATTO, Tokyo, Japan) for BDNF, pERK, pCREB, PSD95; or 5% skim milk (Nacalai Tesque, Kyoto, Japan) for synaptophysin, the membranes were incubated with the following primary antibodies: mature BDNF (the active form of BDNF) (1:200, Merck KGaA, Darmstadt, Germany, #AB1779SP, Host; rabbit), pERK (1:1000, Cell Signaling Technology, Inc., Danvers, MA, U.S.A., #9101S, Host; rabbit), pCREB (1:1000, Cell Signaling Technology, Inc., Danvers, MA, U.S.A., #9198, Host; rabbit), synaptophysin (1:1000, GegeTex, Inc., Irvine, CA, U.S.A., #GTX100865, Host; rabbit), and PSD95 (1:1000, Abcam plc., Cambridge, UK, #ab18258, Host; rabbit). Glyceraldehyde-3-phosphate dehydrogenase (GAPDH) (housekeeping gene) expression was examined as a loading control using an anti-GAPDH antibody (1:1000; Merck KGaA, Darmstadt, Germany, #MAB374, Host; mouse). pERK and pCREB were normalized by total ERK 1/2 (ERK) (1:1000, Proteintech Group, Inc., Rosemont, IL, U.S.A., #16443-1-AP, Host; rabbit) and total CREB (CREB) (1:1000, Cell Signaling Technology, Inc., Danvers, MA, U.S.A., #4820, Host; rabbit), respectively. Next, the membranes were incubated with secondary antibodies (Peroxidase AffinPure Goat Anti-Rabbit IgG (H + L) (Jackson ImmunoResearch, West Grove, PA, U.S.A., #111–035-144) for BDNF, pERK, ERK, pCREB, CREB, synaptophysin, PSD95, and Peroxidase AffinPure Goat Anti-Mouse IgG, F(ab’)2 fragment specific (Jackson ImmunoResearch, West Grove, PA, U.S.A., #115-035-006) for GAPDH), and they were imaged using Western Lightning ECL Pro (PerkinElmer, Waltham, MA, USA) with ImageQuant LAS4000mini (GE Healthcare, Little Chalfont, UK). We quantified the bands using ImageQuant TL software (version 8.1, GE Healthcare, Little Chalfont, UK). Each intensity was normalized to that of GAPDH for BDNF, synaptophysin, or PSD95; to that of ERK for pERK; or to that of CREB for pCREB, and the relative contents were compared to the control group.

### Statistical analysis

All data are presented as mean ± SD. For two-group comparisons, a student’s t-test was used. A one-way analysis of variance (ANOVA) was used to compare the effect of SI without/with the administration of lithium, and a Bonferroni test was used for multiple comparisons. All statistical analyses were performed using EZR (Saitama Medical Center, Jichi Medical University, Saitama, Japan), which is a graphical user interface for R (The R Foundation for Statistical Computing, Vienna, Austria) (Kanda 2013). Differences with *p* values < 0.05 were considered to be statistically significant.

## Results

### FST and EPM after continuous SI in puberty and adulthood

**Fig. 2:**
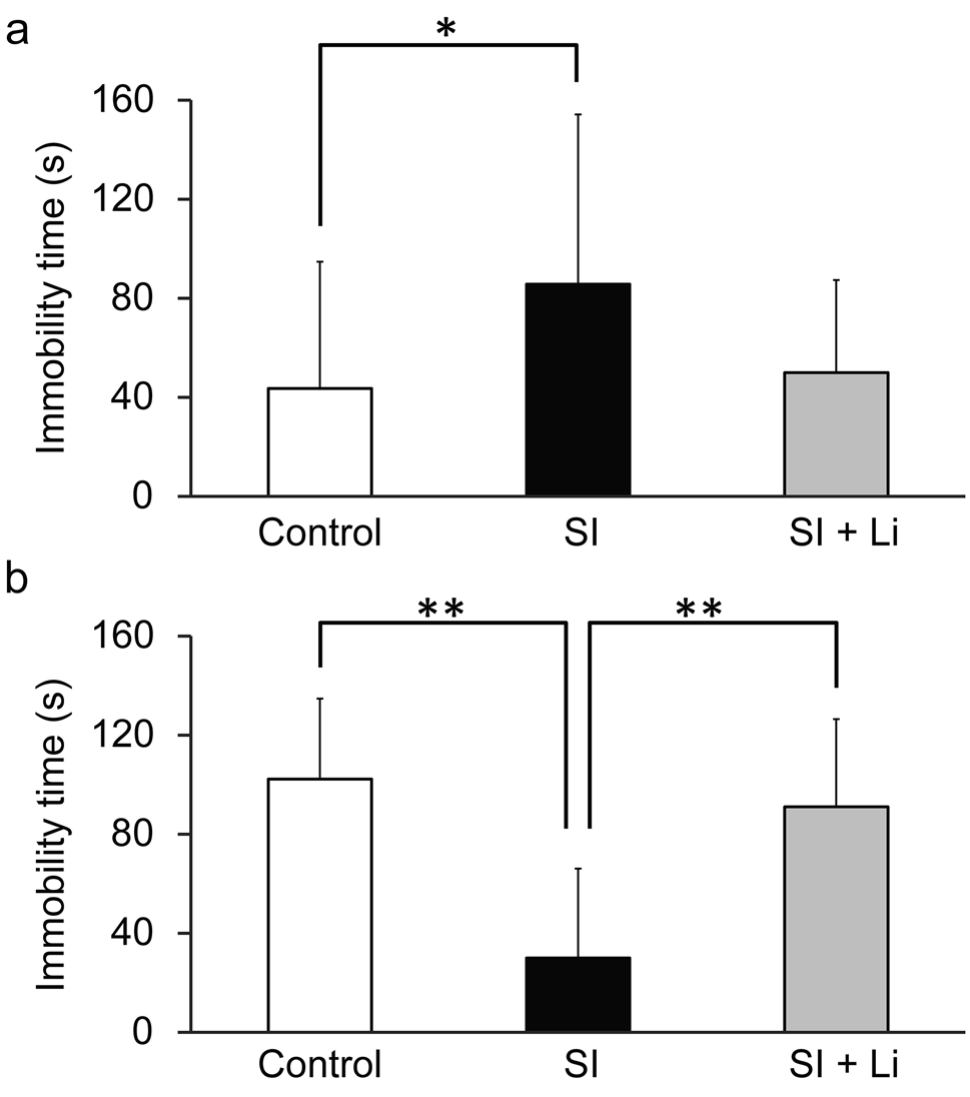
Depressive-like behaviors assessed using the forced swim test after continuous SI in puberty (**a**) or in adulthood (**b**) without/with the administration of lithium (the bar “SI” represents the SI alone group (SI without lithium), and the bar “SI + Li” represents the SI with lithium group). Data are shown as mean ± SD (*n* = 9–21). *p < 0.05, **p < 0.01. SI: social isolation

**Fig. 3:**
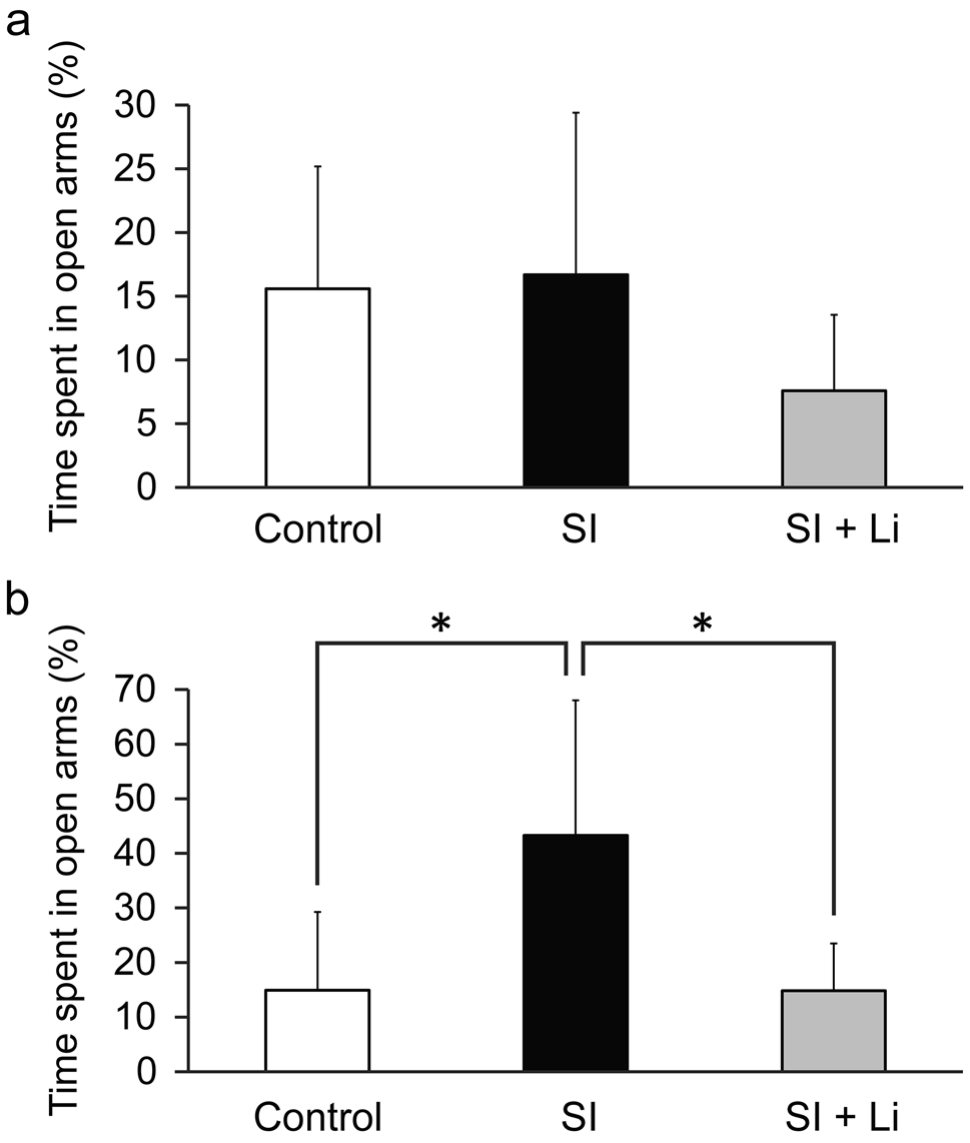
Anxiety-related behaviors assessed using the elevated plus-maze test after continuous SI in puberty (**a**) or in adulthood (**b**) without/with the administration of lithium (the bar “SI” represents the SI alone group (SI without lithium), and the bar “SI + Li” represents the SI with lithium group). Data are shown as mean ± SD (*n* = 7–14). **p < 0.01. SI: social isolation

In the FST, the immobility time of the group of rats in SI until puberty was significantly longer than that of the control group (t(40) = 2.26, *p* = 0.030) (Fig. 2a). However, conversely, the immobility time of the group of rats in SI until adulthood was significantly shorter than that of the control group (t(16) = 4.46, *p* = 0.00039) (Fig. 2b). In the EPM, the time spent in open arms of the group of rats in SI until puberty was not significantly different from that of the control group (Fig. 3a); however, the time spent in open arms in the group of rats in SI until adulthood was significantly longer than that of the control group (less anxiety-related behaviors) (t (13) = 2.66, *p* = 0.020) (Fig. 3b). Regarding the total distance, there was no significant difference between the group of rats in SI until puberty or the group of rats in SI until adulthood and the control group (data not shown).

### FCT following continuous SI in puberty and adulthood

**Fig. 4:**
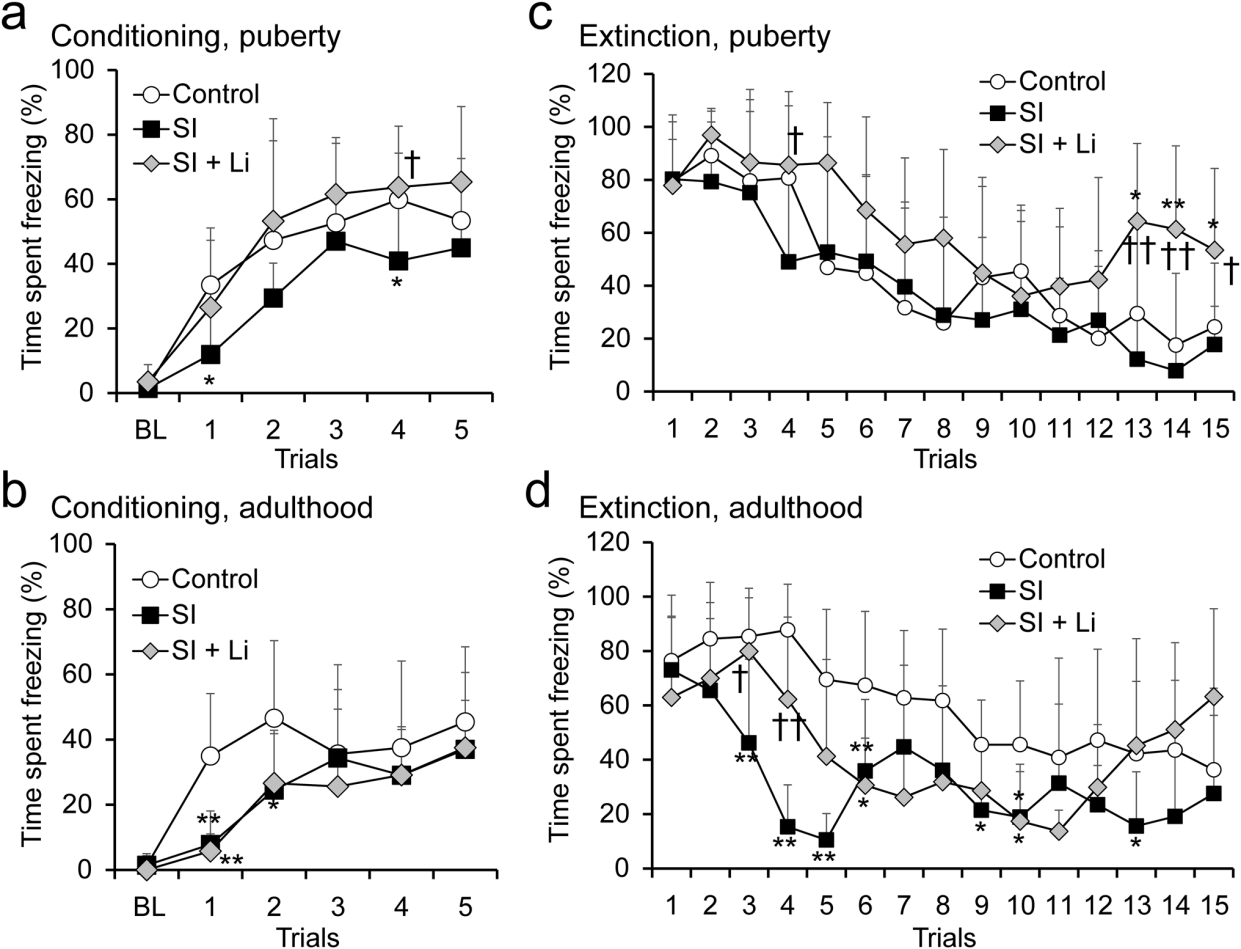
Fear conditioning (**a**, **b**) and extinction (**c**, **d**) assessed using the conditioning fear test after continuous SI in puberty **a**, **c** or in adulthood **b**, **d** without/with the administration of lithium (“SI” represents the SI alone group (SI without lithium), and “SI + Li” represents the SI with lithium group). Data are shown as mean ± SD (*n* = 9–10). *p < 0.05 compared with Control, **p < 0.01 compared with control, ^†^*p* < 0.05 compared with SI (social isolation without lithium), ^††^*p* < 0.01 compared with SI (social isolation without lithium). SI: social isolation, BL: baseline

The time spent freezing the group of rats in SI until puberty tended to be shorter in the conditioning but was not significantly different in the extinction than that of the control group (Fig. 4a, c). The time spent freezing the group of rats in SI until adulthood tended to be shorter in the conditioning and was significantly shorter in the extinction than the control group (Fig. 4b, d).

### The expression of neuronal plasticity-related proteins after continuous SI in puberty and adulthood

**Fig. 5:**
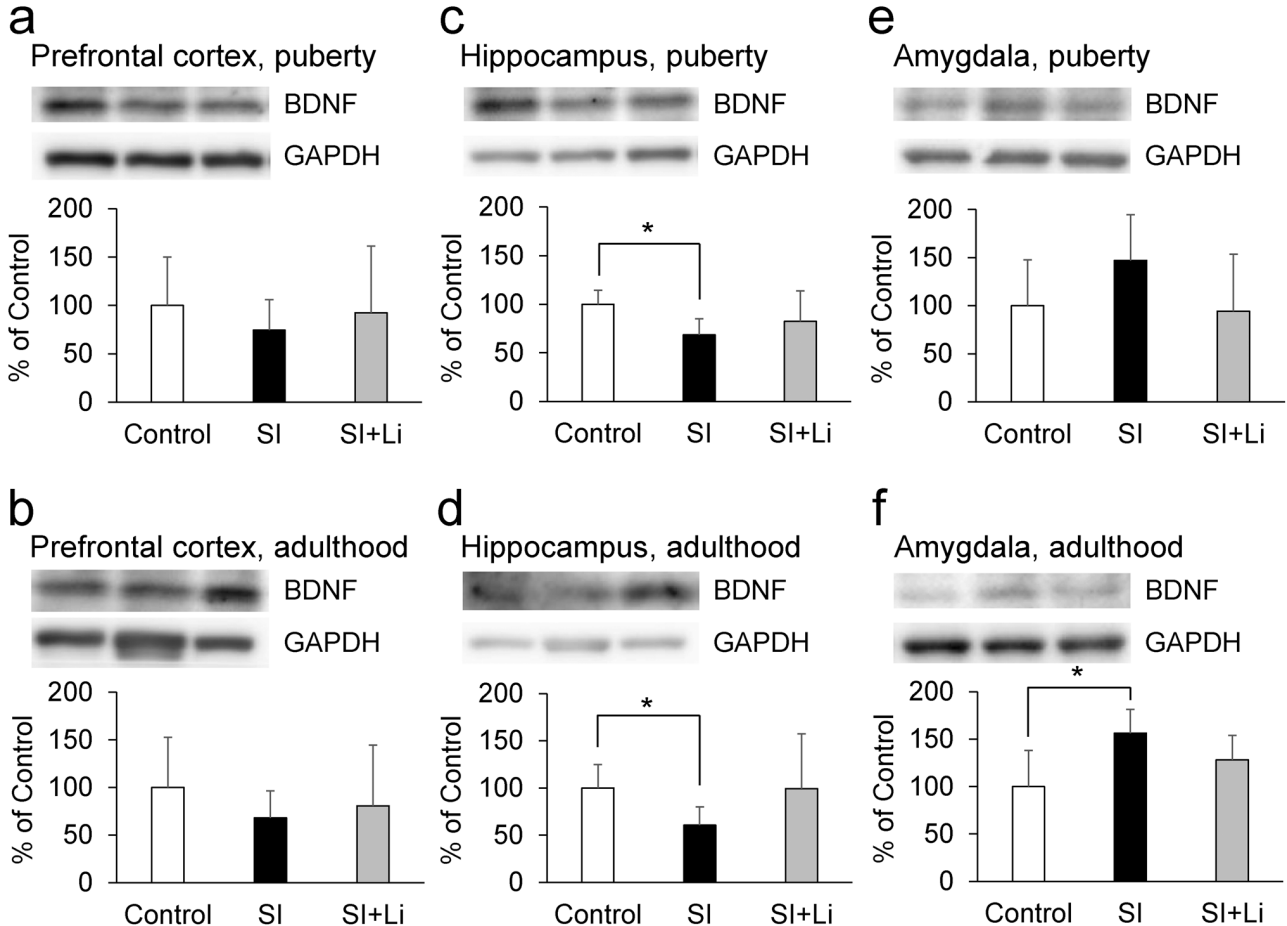
Expression of BDNF after continuous SI in puberty **a**, **c**, **e** or in adulthood **b**, **d**, **f**) in the prefrontal cortex (**a**, **b**), hippocampus (**c**, **d**), and amygdala (**e**, **f**) without/with the administration of lithium (the bar “SI” represents the SI alone group (SI without lithium), and the bar “SI + Li” represents the SI with lithium group). Representative western blot images and relative contents (expressed as a percentage of the control group) are shown. Data are expressed as mean ± SD values (*n* = 4). *p < 0.05. BDNF: brain-derived neurotrophic factor; GAPDH: glyceraldehyde-3-phosphate dehydrogenase; SI: social isolation

**Fig. 6:**
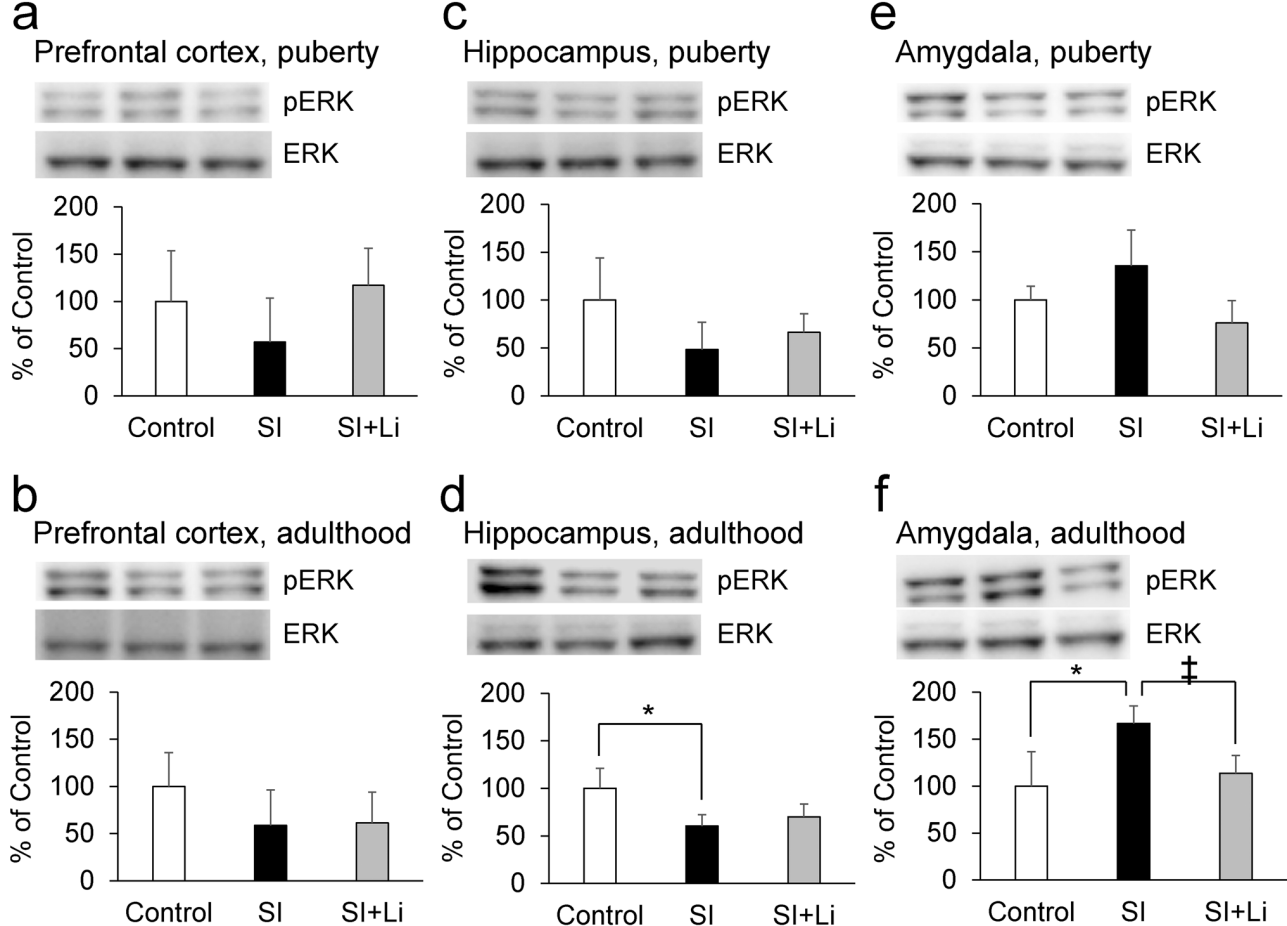
Expression of pERK following continuous SI in puberty **a**, **c**, **e** or in adulthood **b**, **d**, **f** in the prefrontal cortex (**a**, **b**), hippocampus (**c**, **d**), and amygdala (**e**, **f**) without/with the administration of lithium (the bar “SI” represents the SI alone group (SI without lithium), and the bar “SI + Li” represents the SI with lithium group). Representative western blot images and relative contents (expressed as a percentage of the control group) are shown. Data are expressed as mean ± SD values (*n* = 4). *p < 0.05, ‡*p* = 0.055. pERK: phosphorylated extracellular signal-regulated kinase 1/2; ERK: total extracellular signal-regulated kinase 1/2; SI: social isolation

**Fig. 7:**
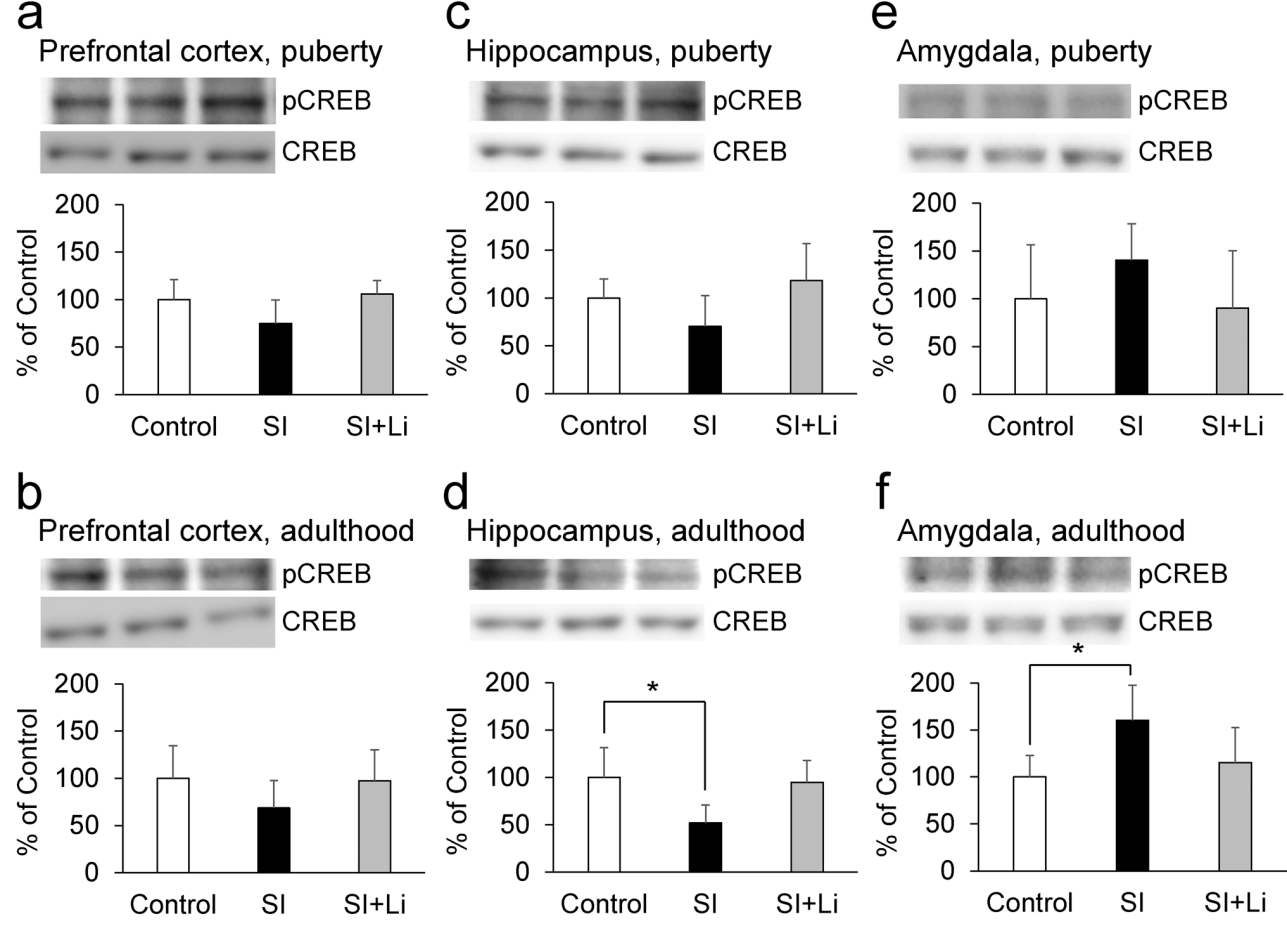
Expression of pCREB after continuous SI in puberty **a**, **c**, **e** or in adulthood **b**, **d**, **f** in the prefrontal cortex (**a**, **b**), hippocampus (**c**, **d**), and amygdala (**e**, **f**) without/with the administration of lithium (the bar “SI” represents the SI alone group (SI without lithium), and the bar “SI + Li” represents the SI with lithium group). Representative western blot images and relative contents (expressed as a percentage of the control group) are shown. Data are expressed as mean ± SD (*n* = 4). *p < 0.05. pCREB: phosphorylated cyclic AMP (cAMP) responsive element binding protein; CREB; total cyclic AMP (cAMP) responsive element binding protein; SI: social isolation

**Fig. 8:**
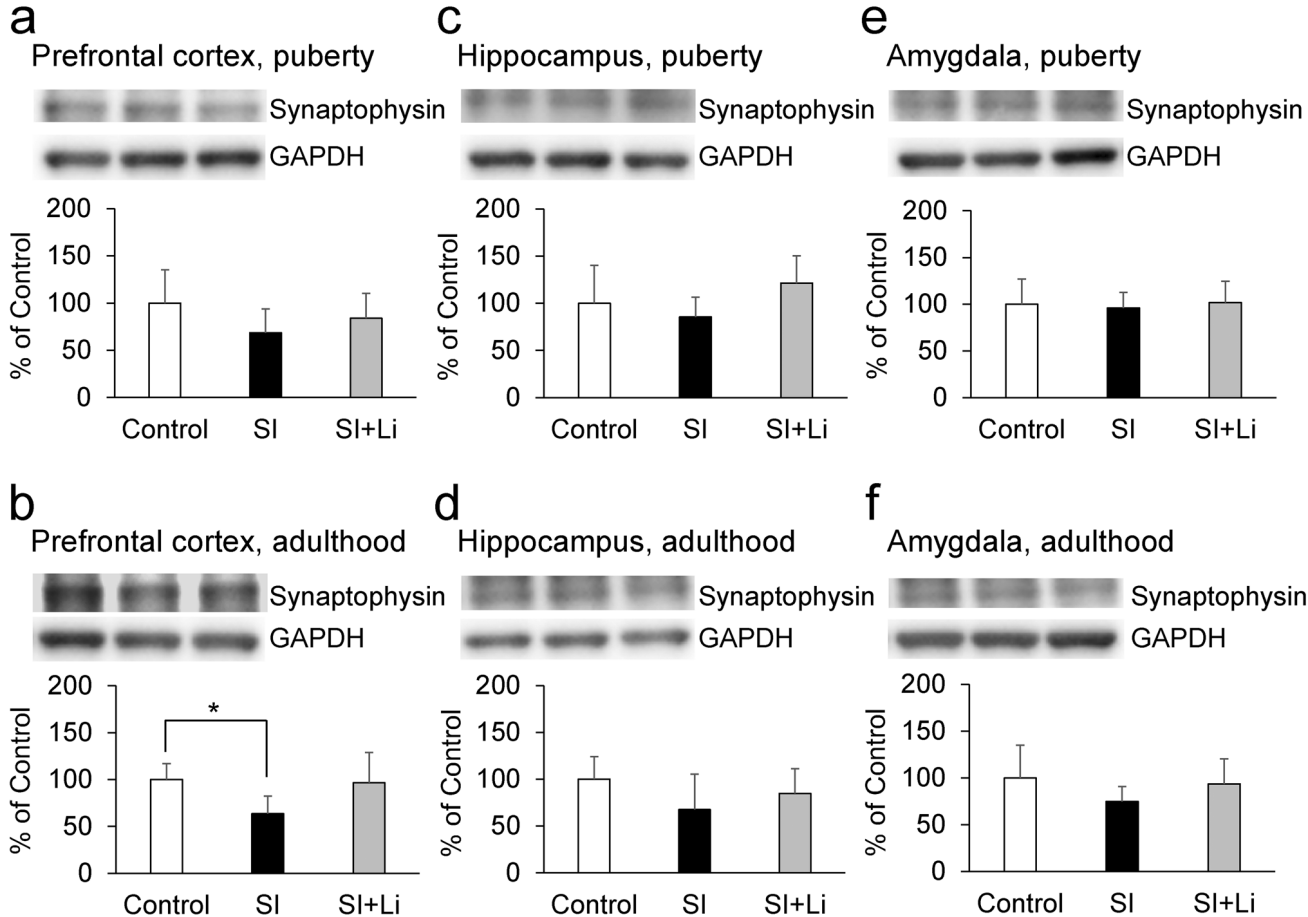
Expression of synaptophysin following continuous SI in puberty **a**, **c**, **e** or in adulthood **b**, **d**, **f** in the prefrontal cortex (**a**, **b**), hippocampus (**c**, **d**), and amygdala (**e**, **f**) without/with the administration of lithium (the bar “SI” represents the SI alone group (SI without lithium), and the bar “SI + Li” represents the SI with lithium group). Representative western blot images and relative content (expressed as a percentage of the control group) are shown. Data are expressed as mean ± SD values (*n* = 4). *p < 0.05. GAPDH: glyceraldehyde-3-phosphate dehydrogenase; SI: social isolation

**Fig. 9:**
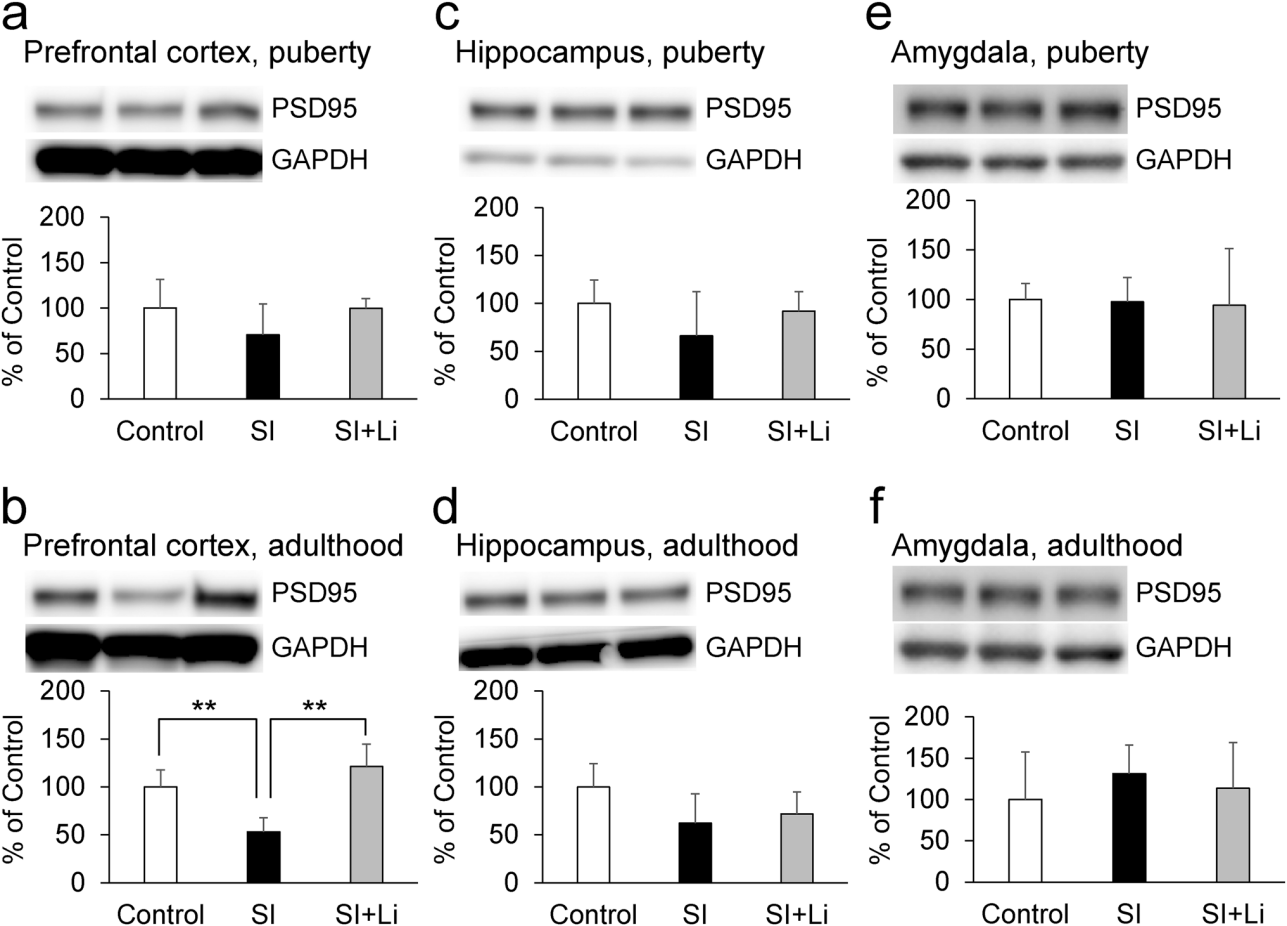
Expression of PSD95 after continuous SI in puberty **a**, **c**, **e** or in adulthood **b**, **d**, **f** in the prefrontal cortex (**a**, **b**), hippocampus (**c**, **d**), and amygdala (**e**, **f**) without/with the administration of lithium (the bar “SI” represents the SI alone group (SI without lithium), and the bar “SI + Li” represents the SI with lithium group). Representative western blot images and relative contents (expressed as a percentage of the control group) are shown. Data are expressed as mean ± SD (*n* = 4). **p < 0.01. PSD95: post-synaptic density 95; GAPDH: glyceraldehyde-3-phosphate dehydrogenase; SI: social isolation

The hippocampal expression of BDNF was significantly lower in the group of rats in SI until puberty compared to the control group (t(6) = 2.87, *p* = 0.029) (Fig. 5c). Changes in the expression of neuronal plasticity-related proteins were more frequent in the group of rats in SI until adulthood compared to the control group in the hippocampus and the prefrontal cortex. Hippocampal BDNF, pERK, and pCREB expression were significantly lower than that of the control group (t(6) = 2.50, *p* = 0.047; t(6) = 3.26, *p* = 0.017; t(6) = 2.58, *p* = 0.042, respectively) (Figs. 5d, 6d, 7d). In the prefrontal cortex of the group of rats in SI until adulthood, the expression of synaptophysin and PSD95 was significantly lower than that of the control group (t(6) = 2.86, *p* = 0.029; t(6) = 4.05, *p* = 0.0067, respectively) (Figs. 8b, 9b). On the other hand, in the amygdala, there was no significant difference in the expression for any of the target proteins in the group of rats in SI until puberty compared to those in the control group. However, in the group of rats in SI until adulthood, the expression levels of BDNF, pERK, and pCREB were significantly higher than those of the control group (t(6) = − 2.49, *p* = 0.047; t(6) = − 3.25, *p* = 0.017; t(6) = − 2.76, *p* = 0.033, respectively) (Figs. 5f, 6f, 7f).

### The effect of lithium on FST and EPM after continuous SI in puberty and adulthood

In the FST, no significant difference in immobility time was noted in the lithium-administered rats in SI until puberty or the lithium-administered rats in SI until adulthood compared to the control group (Fig. 2). Furthermore, the immobility time of the lithium-administered rats in SI until adulthood was significantly longer than that of non-lithium-administered rats in SI until adulthood (F(2, 25) = 11.37, *p* = 0.0024) (Fig. 2b). Similar to the FST results, in the EPM, there was no significant difference in the time spent in open arms for the lithium-administered rats in SI until puberty or the lithium-administered rats in SI until adulthood compared to those in the control group (Fig. 3). The lithium-administered rats in SI until puberty tended to display shorter times spent in open arms compared to the control group or group of non-lithium-administered rats in SI, but the differences were not significant (F(2, 34) = 2.41; *p* = 0.23, 0.14, respectively) (Fig. 3a). Furthermore, the time spent in the open arms for the lithium-administered rats in SI until adulthood was significantly shorter than that of the non-lithium-administered rats in SI until adulthood (more anxiety-related behaviors) (F(2, 20) = 6.96, *p* = 0.011) (Fig. 3b). Regarding the total distance, there was no significant difference between the lithium-administered and non-lithium-administered rats in SI or control group in puberty and adulthood (data not shown).

### The effect of lithium on FCT following continuous SI in puberty and adulthood

The time spent freezing the lithium-administered rats in SI until puberty was not significantly different from that of the control group in both the conditioning and extinction trials, except for the last three extinction trials (Fig. 4a, c). On the other hand, the time spent freezing the lithium-administered rats in SI until adulthood tended to be shorter than that of the control group in both the conditioning and extinction trials but longer than that of the group of rats not administered lithium in the extinction trial (Fig. 4b, d).

### The effect of lithium on the expression of neuronal plasticity-related proteins after continuous SI in puberty and adulthood

As shown in Fig. 5–9, there was no significant difference in the expression for any of the target proteins in the prefrontal cortex, hippocampus, or amygdala in the group of lithium-administered rats in SI until puberty, or in the group of lithium-administered rats in SI until adulthood, compared to those in the control group. Furthermore, in the amygdala, the expression of pERK in the lithium-administered group of rats in SI until adulthood tended to be lower (F(2, 9) = 7.27, *p* = 0.055) than that of the group of rats not administered lithium (Fig. 6f). In the prefrontal cortex, the expression of PSD95 in the lithium-administered group of rats in SI until adulthood was significantly higher than that of the group of rats not administered lithium (F(2, 9) = 13.42, *p* = 0.0020) (Fig. 9b).

## Discussion

To the best of our knowledge, this report is the first to directly compare the influence of continuous SI after weaning on depressive-like and anxiety-related behaviors and neuronal plasticity damage in puberty and adulthood. In the behavioral tests, the immobility time in the FST of the group of rats undergoing SI until puberty was significantly longer than that of the control group. This suggests that SI from early age-induced depressive-like behaviors in puberty, as previously reported (Raineki 2012). Conversely, in the group of rats exposed to SI until adulthood, the immobility time in the FST was significantly shorter and the time spent in the open arms in the EPM was significantly longer than in the control group. Thus, with SI from an early age into adulthood, depressive-like behaviors did not increase, but, rather, paradoxically decreased, and these data of FST and EPM in adulthood are thought to complement each other. Notably, the method of SI was identical throughout the whole experiment. This suggests that the extension of the duration of SI from an early age does not simply worsen depressive-like behaviors, but, instead, induces another type of behavior. In comparison with periods of puberty and adulthood for the control group, the time spent in immobility in the FST increased from about 40 s to 100 s, but the time spent in open arms in the EPM was nearly the same. Therefore, development may impact depressive-like behaviors rather than anxiety-related behaviors. Furthermore, the time spent in open arms of the EPM in the group of rats in SI until puberty was not significantly different compared to the control group. It has previously been reported that young rats are less affected by anxiogenic stimuli compared to adult ones because their anxiety-regulating brain mechanisms are still immature (Doremus et al. 2004). Therefore, the uncertainty surrounding anxiety-related behaviors in puberty in this study may also be related to this immaturity.

Unlike our results, many previous studies have revealed the induction of depressive-like or anxiety-related behaviors in rats in adulthood after post-weaning SI (3–6). In contrast, it is reported that early handling, as was performed in this study, alters rats’ behavioral stress reactivity in adulthood (Meerlo et al. 1999). In particular, male handled rats had shorter immobility times in the FST after chronic stress (Papaioannou et al. 2002). Our study used male rats too. The anxiogenic effects of SI could also be eliminated by handling rats only a few times per week (Holson et al. 1991). Furthermore, early handling—along with SI—reduced fear and induced risk-taking behavior (Spivey et al. 2008). Therefore, handling performed during breeding in this study may have contributed to the behavioral changes observed in adulthood. Furthermore, other differences in the experimental design, such as the timing to start SI or the size of the breeding cage, may explain the inconsistent outcomes between our study and previous reports (Ravenelle et al. 2014; Arakawa 2018). Several studies have reported that post-weaning SI did not influence depressive-like or anxiety-related behaviors. For example, after SI in 3-week-old rats for 6 weeks, there was no significant difference in the immobility time of the FST (Soga et al. 2015). Nine weeks (P63) correspond to an age between puberty (P42) and adulthood (P77) as defined in our study. Therefore, it is possible that depressive—or anxiety-related behaviors were not observed in these studies, as behavioral tests were performed in a period between the induction and reduction of these behaviors.

The relationship between SI and depression has been well investigated. The indicators of SI are associated with depressive symptoms (Ge et al. 2017). The presence of SI is useful in identifying college students in need of mental health services for depressive symptoms (Hill et al. 2015). SI also reduces the efficacy of general treatments for depression (Brown et al. 2011). In puberty or adolescence, depression can be caused by SI from an early age (Ford and Rechel 2012; Shaw and Dallos 2005). In adulthood, SI experienced at an early developmental stage is also a risk factor for subsequent depression (Riise and Lund 2001), but environmental enrichment after SI can counteract the symptoms of depression (Simonds et al. 2014). However, people who were unable to socially recover and were exposed to long-term SI or neglect from an early age may develop other psychiatric problems such as violence or aggression (Kalvin and Bierman 2017; Naugton et al. 2013). This study implies that these changes from puberty or adolescence to adulthood may occur due to continuous SI alone, even if other stresses are not added on.

In the FCT, the time spent freezing in the group of rats in SI until puberty tended to be shorter than that of the control group in the conditioning. This suggests that SI from early age tended to decrease fear-related behaviors in puberty. Furthermore, the time spent freezing in the group of rats in SI until adulthood tended to be shorter in the conditioning and was significantly shorter in the extinction compared to the control group. Thus, with SI from an early age into adulthood, fear-related behaviors decreased more significantly. Many previous studies have revealed that chronic stress increases fear-related behaviors (Zhang and Rosenkranz 2013) (Guadagno et al. 2017). On the other hand, several groups have reported a stress-induced decrease in fear-related behaviors, as in this study (Brydges et al. 2014) (Gresack et al. 2010). Voikar V et al. suggested that the reduced freezing after long-term individual housing could be explained by attempts to avoid or escape the arena in addition to the possible memory deficit (Voikar et al. 2005). These factors may also be related to the decrease in fear-related behaviors after SI in this study. As mentioned above, depressive-like behaviors in the FST and EPM of this study revealed paradoxical changes along with the SI from an early age into puberty or adulthood. However, in the FCT, a decrease in fear-related behaviors became more prominent with the extension of the duration of SI. Therefore, it is suggested that the mechanisms underlying the behavioral changes observed in the FST and EPM are different from those observed in the FCT.

Regarding the neuronal plasticity-related proteins, SI until puberty decreased the expression of BDNF, and SI until adulthood decreased the expression of pERK and pCREB in addition to BDNF in the hippocampus. Furthermore, SI until puberty did not change the expression of synaptophysin and PSD95, but SI until adulthood decreased their expressions in the prefrontal cortex. Therefore, the extension of the duration of SI from an early age accentuated the changes in neuronal plasticity-related protein expression in the hippocampus and the prefrontal cortex, although depressive-like behaviors observed in puberty were paradoxically decreased in adulthood. The mitogen-activated protein kinase (MAPK)/ERK signaling cascade serves for the stimulus-induced transcription, and pERK is an active form of ERK (Jia et al. 2013). CREB, a transcription factor downstream of ERK (Licznerski and Duman 2013), is activated by the phosphorylation of the serine 133 residue (Shaywitz and Greenberg 1999), and pCREB induces the transcription of BDNF by binding to the cAMP/Ca(2 +)-response element (CRE) in the BDNF promoter (Pruusnild 2011). Furthermore, BDNF promotes the phosphorylation of ERK and CREB by creating a positive feedback loop through tyrosine kinase receptor B (TrkB) and the MAPK/ERK signaling cascade (Davis et al. 2000; Gourley et al. 2008). Therefore, it was suggested that SI until puberty decreased the amount of BDNF, and, after the extension of SI until adulthood, pERK and pCREB in addition to BDNF was decreased by the inhibition of that positive feedback loop. The basal expressions of synaptophysin and PSD95, which is associated with pre-synaptic and post-synaptic plasticity and maturation respectively, is reported to be higher in the adult central nervous system than in the juvenile one (Chang et al. 2020; Vickers et al. 2006). Therefore, it is thought that synaptophysin and PSD95 are more critical to neuronal function in adulthood than in puberty and that the effect of continuous SI from an early age may be amplified in adulthood. On the other hand, SI until adulthood increased the expression of BDNF, pERK, and pCREB in the amygdala. It has been reported that chronic stress induces hypertrophy and hyperactivity of amygdala (Vyas et al. 2002; Rosenkranz et al. 2010). The amygdala plays an important role in the expression of affect, especially as related to fear (Zhang and Rosenkranz 2013). Therefore, these different patterns of expression of BDNF, pERK, and pCREB in the hippocampus and amygdala may contribute to the different patterns of depressive-like behaviors in the FST and EPM and fear-related behaviors in the FCT.

After the administration of lithium, neither increase in depressive-like behaviors in puberty nor a decrease in depressive-like and anxiety-related behaviors in adulthood was observed. Furthermore, lithium reversed the decrease in the expression of neuronal plasticity-related proteins in both puberty and adulthood in the prefrontal cortex and hippocampus. Therefore, lithium may improve these behavioral changes seen in puberty and adulthood after continuous SI from an early age by reversing the damage incurred to neuronal plasticity in these brain regions. Previously, we described that a moderate impairment of neuronal plasticity induces a depressive-like state; however, further impairment does not increase depression, but rather causes a manic-like state (Mizuno et al. 2013). This possibility is consistent with the fact that lithium exerts both anti-depressive and antimanic effects as a mood stabilizer, and that lithium has a neurotrophic effect (Won and Kim 2017; Kessing et al. 2018). Furthermore, these results may imply that lithium may be effective in the treatment of unstable mood linked to poor environmental conditions from an early age. On the other hand, in this study, the effect of lithium to improve behavioral changes following SI is clearly observed in adulthood. Furthermore, behavioral improvements by lithium in adulthood were observed without improving the levels of BDNF, pERK, and pCREB in the hippocampus, even though there was no significant difference in the expression of these proteins in the group of lithium-administered rats in SI until adulthood, as compared to the control group. However, in the prefrontal cortex, the expression of PSD95 in the group of lithium-administered rats in SI until adulthood (not until puberty) was significantly higher than that of the group of rats not administered lithium. Therefore, especially in adulthood, PSD95 in the prefrontal cortex may contribute more to the behavioral improvement after SI compared to BDNF, pERK, and pCREB in the hippocampus. After the administration of lithium, body weight was decreased in both puberty and adulthood, as previously reported (Ramanadan et al. 2012); however, in the EPM, the administration of lithium did not influence the total distance in either puberty or adulthood. Therefore, the effect of lithium observed in this study is thought to be mood-related, rather than activity-related. In the FCT, following the administration of lithium, the decrease in fear-related behaviors in puberty was not observed in the conditioning or the extinction, and the decrease in fear-related behaviors in adulthood was less significant in the conditioning compared to the group of rats not administered lithium. Furthermore, lithium reversed the changes in the expression of neuronal plasticity-related proteins in both puberty and adulthood. Therefore, lithium may also improve the changes in fear-related behaviors seen in puberty and adulthood following continuous SI from an early age, remodeling the changes incurred on neuronal plasticity.

Several issues remain to be resolved. The numbers of animals in each group are not even because of experimental circumstance. In this study, the effect of SI from an early developmental stage was evaluated in puberty and adulthood using male rats, but its effect at other stages, such as in old age or in female rats, remains unclear. Dysfunction in neuronal plasticity was assessed by examining the expression of neuronal plasticity-related proteins, but additional methods, such as the representative electrophysiological readings for LTP/LTD or the evaluation of spine sizes and puncta count, are warranted to confirm our results. The changes in the regional expression patterns of neuronal plasticity-related proteins differed between the prefrontal cortex and hippocampus. The mechanisms through which SI induces neuronal plasticity dysfunction may vary across each brain region, and assessing expression patterns in more brain regions will be important. While this is a basic study using experimental animals, in the future, we hope to develop our research to include human studies comparing the effects continuous SI from an early developmental age on mood at various stages.

## Conclusion

Continuous SI after weaning increased depressive-like behaviors in puberty and decreased depressive-like and anxiety-related behaviors in adulthood. In contrast, in the prefrontal cortex and hippocampus, changes in neuronal plasticity-related protein expression observed in puberty gained prominence in adulthood. Therefore, it is suggested that the extension of the duration of SI from an early developmental stage does not simply worsen depressive-like behavior, but, rather, induces another type of behavior associated with substantial changes in protein expression of these brain regions. In contrast, SI after weaning decreased fear-related behaviors with the increase of neuronal plasticity-related protein expression in the amygdala. After the administration of lithium, no behavioral changes or changes in neuronal plasticity-related protein expression in puberty and adulthood were observed. Therefore, lithium may improve the behavioral changes in puberty and adulthood after continuous SI from an early developmental stage by reversing damage incurred to neuronal plasticity. The mechanisms underlying the depressive-like and anxiety-related behaviors may be different from those underlying the fear-related behaviors.

## Data Availability

The datasets generated and analyzed during the current study are available from the corresponding author on reasonable request.
